# Evaluation of safety and efficacy of a 20% Subcutaneous Immunoglobulin (Hizentra™), after a dose equivalent switch from intravenous or subcutaneous replacement therapy in a cohort of primary immunodeficient patients

**DOI:** 10.1186/1710-1492-8-S1-A19

**Published:** 2012-11-02

**Authors:** Adriana Martin, Robert Schellenberg

**Affiliations:** 1 SCIG Home Infusion Program, St. Paul’s Hospital, Vancouver, British Columbia, Canada

## Objectives

To assess the safety and efficacy of Hizentra™, a 20% human IgG for subcutaneous administration, in a cohort of patients with primary immunodeficiency disorders (PID) after a dose equivalent switch from their previous treatment.

## Methods

A cohort study of 57 PID patients was reviewed 3 months post-transition to the 20% Subcutaneous Immunoglobulin (Hizentra™), in order to evaluate clinical outcomes and adverse events related to a dose-equivalent switch from 10% liquid solution intravenous (Privigen® and Gamunex®) or 16% subcutaneous replacement therapy (Vivaglobin®) to weekly infusion of Hizentra™, Descriptive analyses were performed in relations to IgG levels, total infusion volume and infusion time.

## Results

Mean age of patients was 51.8 years old. The study showed IgG levels achieved with Hizentra™, were similar to pre-study levels with subcutaneous and higher by 17.1% compared to intravenous IgG. Local reactions were minimal and only one Hizentra™-related adverse event was reported. Generally, lower infusion volume with Hizentra™, also led to a reduction in total infusion time and in the number of infusion sites compared to other subcutaneous replacement therapy.

## Conclusions

Switching to Hizentra™, maintained serum IgG levels without dose increases, overall reduction in infusion time and infusion sites. It therefore confirms that for our cohort, this new strategy is practical while being similar in effectiveness and safety to both intravenous and subcutaneous replacement therapy.

